# Mental health and nature connectedness. A step toward a new mental health definition?

**DOI:** 10.1192/j.eurpsy.2025.2001

**Published:** 2025-08-26

**Authors:** M. Gawrych

**Affiliations:** The Maria Grzegorzewska Univeristy, Warsaw, Poland

## Abstract

**Introduction:**

Recent studies have shown the mental health is broad concept. Nature connectedness seems to be one of important factors affecting it.

**Objectives:**

to clarify the concept of mental health in term of nature connectedness.

**Methods:**

The MEDLINE database (through PubMed) was searched for articles in this field published in 2010–2024. Mental health-related descriptors (i.e., “mental health” or “mental disorders”) and the “nature/ social connectedness” terms were used in particular searches.

**Results:**

The selected publications were categorized and then analysed. Strong nature connectedness is associated with higher mental well-being. Full results will be presented.

**Conclusions:**

Nature connectedness can play a crucial role in understanding some aspects of mental health as well as mental health prevention.

**Disclosure of Interest:**

None Declared

